# Inhibition of chronic prostate inflammation by hyaluronic acid through an immortalized human prostate stromal cell line model

**DOI:** 10.1371/journal.pone.0178152

**Published:** 2017-05-30

**Authors:** Ming-Che Liu, Wei-Hong Chen, Chi-Sheng Chiou, Wen-Cheng Lo, Navneet Kumar Dubey, Yu-Chin Chen, Wen-Fu T. Lai, Shauh-Der Yeh, Han-Sun Chiang, Win-Ping Deng

**Affiliations:** 1 Department of Urology, School of Medicine, College of Medicine, Taipei Medical University, Taipei, Taiwan, R.O.C; 2 Department of Urology, Taipei Medical University Hospital, Taipei, Taiwan, R.O.C; 3 Graduate Institute of Biomedical Materials and Tissue Engineering, College of Biomedical Engineering, Taipei Medical University, Taipei, Taiwan, R.O.C; 4 Stem Cell Research Center, Taipei Medical University, Taipei, Taiwan, R.O.C; 5 School of Dentistry, College of Oral Medicine, Taipei Medical University, Taipei, Taiwan; 6 Division of Allergy, Immunology and Rheumatology, Department of Internal Medicine, Taipei Medical University Hospital, Taipei, Taiwan, R.O.C; 7 Department of Neurosurgery, Taipei Medical University Hospital, Taipei, Taiwan, R.O.C; 8 School of Medicine, Taipei Medical University, Taipei, Taiwan; 9 Graduate Institute of Clinical Medicine, Taipei Medical University, Taipei, Taiwan; 10 Institute of Biomedical Sciences, Academia Sinica, Taipei, Taiwan; 11 Division of Urology, Department of Surgery, Cardinal Tien Hospital, Taipei, Taiwan; 12 College of Medicine, Fu-Jen Catholic University, Taipei, Taiwan; 13 College of Oral medicine, Taipei Medical University, Taipei, Taiwan; Florida State University, UNITED STATES

## Abstract

Benign prostatic hyperplasia (BPH) is the most common urologic disease among elderly men. A well-established *in vitro* cell model is required to determine the therapeutic mechanism of BPH inflammation. In this study, we attempted to establish an immortalized human prostate stromal cell line by transfecting with HPV-16 E6/E7 and designated as ihPSC. No significant difference was found in fibroblast-like morphology between primary hPSC and ihPSC. The ihPSC possessed a significantly higher cell proliferation rate than primary hPSC. The prostate-specific markers and proteins including cytoskeleton (α-SMA and vimentin) and smooth muscle (calponin), especially the androgen receptor (AR) were also examined in ihPSC, almost identical to the primary hPSC. To create an *in vitro* model featuring chronic prostatic inflammation, ihPSC was stimulated with IFN-γ+IL-17 and then treated with the high molecular weight hyaluronic acid hylan G-F 20 as an alternative strategy for inhibiting BPH inflammation. Hylan G-F 20 could dose-dependently diminish the inflammation-induced proliferation in ihPSC. The enhanced expressions of inflammatory molecules including IL-1β, IL-6, IL-8, cyclooxygenase 2 (COX2), inducible nitrogen oxide synthase (iNOS), and Toll-like receptor 4 (TLR4) were all abolished by hylan G-F 20. For inflammatory signaling, hylan G-F 20 can also diminish the IFN-γ+IL-17-increased expression of iNOS and p65 in ihPSC. These findings suggest that ihPSC could provide a mechanism-based platform for investigating prostate inflammation. The hylan G-F 20 showed strong anti-inflammatory effects by decreasing inflammatory cytokines and signalings in the ihPSC, indicating its therapeutic potentials in BPH treatment in the future.

## Introduction

Benign prostatic hyperplasia (BPH) represents the most common urologic disease among elderly men, in which the incidence is over 70% at age 60 years and over 90% at age 70 years [1, 2]. There is increasing evidence for the association of chronic prostate inflammation with BPH [3–5]. Inflammation in BPH tissue includes the up-regulation of pro-inflammatory cytokines such as IL-17 in infiltrating T cells [6], interferon-γ in basal and stromal cells [7], and IL-8 in epithelial cells [8]. A variety of growth factors and cytokines have also been implicated in BPH inflammation, such as IL-1, IL-6, IL-8, and IL-17 as well as TNF-α and TGF-β [9]. In addition, preventing or reducing prostate inflammation might be one strategy for reducing the risk of prostate cancer (PC) and therefore targeting inflammation sources is considered as an attractive option. Hence, therapeutic strategy of targeting the prostate stoma, especially the prostate stromal cells, has become emerged.

An *in vitro* cell model is required for preclinical study to determine the mechanism of BPH inflammation. Unfortunately, primary human prostate cells are known very difficult to be developed for continuously growing culture and undergo terminal growth arrest [10]. The differentiation state also rapidly loses following *in vitro* culture. Thus, an immortalized prostate cell lines with innate and stable characteristics is indispensable for BPH research. Various approaches have been reported to reach immortalization, including the transfection of telomerase reverse transcriptase (TERT) and oncogene SV40LT into parental cells. However, disadvantages such as karyotypic instability and cell hypertrophy were commonly realized [11, 12]. To obtain immortalized cell lines retaining innate and parental phenotypes, *human papillomavirus-16 E6/E7* (*HPV-16 E6/E7*) has been successfully utilized in our previous studies [13–15]. The preservation of parental characteristics, differentiation abilities, and unlimited cell proliferation were elucidated after *HPV-16 E6/E7*-immortalization. Thus, it is feasible to use *HPV-16 E6/E7* for immortalizing human prostate stromal cells.

Another critical issue is to develop *in vitro* BPH inflammation model. The infiltration of immune cells including T cells, B cells, and macrophages has been demonstrated in contributing BPH formation [16]. Most importantly, IFN-γ and IL-17 secreted by CD4+ cells could up-regulate IL-6, IL-8, and CXCL10 production in BPH cells and create a positive feedback loop for enhancing BPH inflammation [17]. Thus, IFN-γ and IL-17 were cooperatively used to create *in vitro* BPH inflammatory model on ihPSC.

Considering the importance of the stromal elements in the development and progression of BPH, the present study was aimed to create an immortalized human prostate stromal cell (designated as ihPSC) model by employing the human papillomavirus type 16 (HPV16) E6/E7 gene. The phenotypes and growth profile of this ihPSC cell line was further verified to evaluate its potential for functional studies and for prospective applications, such as a screening tool to identify potential agents with anti-inflammatory activities. For BPH treatment, high molecular weight-hyaluronic acid (HMW-HA) with strong anti-inflammation potentials was utilized to explore its molecular mechanism for anti-inflammation and for future therapy by using the ihPSC model.

## Material and methods

### Primary culture of prostate stromal cells

The study protocol was approved by the Joint Institutional Review Board at the Taipei Medical University, Taiwan (TMUH-JIRB 103-01-R1). Specimens were collected by transurethral resection of the prostate (TURP) from patients who signed an informed consent to the approved study protocol. Histology of respected specimens was confirmed by pathological report from a surgical pathologist in which a benign inflammatory prostate tissue with proliferation of prostatic acini and fibromuscular stroma were evident.

Primary human prostate stromal cells (hPSC) were isolated from specimens with histological diagnosis within 4 h of resection. Tissues were transferred to sterile vessels in growth medium containing DMEM/F12 (1:1) medium (Invitrogen) supplemented with 10% fetal bovine serum (FBS), 2mM L-Glutamine and antibiotics) and finely chopped using scissors. Suspensions containing tissue fragments were then digested at 37°C in 200 IU/ml type I collagenase (Sigma) for 18 hours. The collagenase digested tissue was then washed three times in PBS and the supernatant containing stromal cells was centrifuged at 250×g (or 500 rpm) for 5 minutes. Cell pellet was re-suspended and plated in growth medium and incubated at 37°C in a 5% CO_2_/humidified incubator. After 12 hours, non-adherent material was removed by washing with fresh medium. After the cells were grown to confluence, stromal cells were harvested by trypsinization and then removed and expanded under routine conditions. After cultivation for 2 passages, a homogenous stromal cell population was established.

### Immortalization of primary hPSC by transfecting with human papillomavirus (HPV-16)-E6/E7

Transduction of the *HPV-16 E6/E7* was conducted as previously described [15, 18]. In brief, the *HPV-16 E6/E7* retroviral vector (LXSN16E6E7) [18, 19] produced by PA317 cell line (purchased from the American Type Culture Collection, ATCC, Manassas, VA, USA) was expanded in DMEM with 10% FBS (Gibco). The isolated primary polyclonal human prostate stromal cells (hPSC) were recovered by trypsinization, and then seeded on six-welled plates at a density of 2.5 x 10^5^ cells per well. After infection with 1mL virus stock in medium containing 8 μg/mL polybrene for 48 hours, the virus was removed and the medium was replaced with DMEM/F12 supplemented with 10% FBS. The cells were passaged on the next day and harvested when obvious clones were present three weeks later. The transfected cells, designated as ihPSC, were continuously grown in the same medium as used for primary culture and passaged at a ratio of 1:3 when the cells appeared subconfluently.

### Cell growth assay

The cell viability was estimated using MTT assay (Sigma, USA), which provides a relative measure of cell growth by quantifying cellular conversion of a tetrazolium salt into a formazan product. Cells (4 × 10^3^ cells/ well) were seeded into 96-well plates and allowed to grow cultured for different times. The growth rate was determined in triplicate on days 1, 3, 5, and 7 after inoculation, respectively. MTT reagent (20 μL/ well) was added and incubated for another 4 hours at 37°C in a 5% CO_2_ incubator. 150 μL of DMSO (Sigma, USA) was added to each well to lyse cells and dissolve the crystals. Viable cells were determined by reading the absorbance of the cell lysates at 570 nm by using a Multiskan PC (Thermo Labsystem). Cell population doubling time was calculated using the following function as previously described [18]:

$$Doubling\;time=\frac{(T-T_{0})\;log2}{logN-logN_{0}}$$

where T–T_0_ indicates the length of time between two measurements and N_0_ and N denote the OD value at the initial seeding time point and the final time point. The experiments were repeated three times.

### Senescence-associated β-galactosidase (SA-β-gal) staining

Cell senescence was characterized by changes in SA-β-gal activity and performed using a histochemical staining kit (Sigma). Briefly, hPSC and ihPSC cells were incubated with fixation buffer for 6–7 minutes at room temperature. After washing with PBS, the cells were incubated with β-gal chromogenic substrate solution for 12 h at 37°C without CO_2_. PBS was then added to stop the reaction and the stained cells were counted. The experiment was repeated times and the mean percentage of cells expressing β-Gal was calculated.

### Reverse transcriptase polymerase chain reaction (RT-PCR)

Total RNA was extracted using TRIzol reagent (Invitrogen Life Technologies, Carlsbad, CA) according to the manufacturer’s instructions. Extracted RNA was quantified spectrophotometrically at 260nm, dissolved in sterilized ddH_2_O and stored at −80°C until use. Reverse transcription (RT) was performed with SuperScript^TM^ III Reverse Transcriptase (Invitrogen Life Technologies) and an Oligo d(T)_12-18_ primer according to manufacturer’s instructions. Six micrograms of cDNA was used in PCR amplification in a final volume of 50 μL containing 2.5 mM dNTP, 25 mM MgCl_2_, upstream/downstream primers (see Table 1) and Taq DNA polymerase (Invitrogen Life Technologies). Following an initial denaturation at 95°C for 5 min, the DNA was amplified in the Touchgene Gradient (Techne, Cambridge, UK) using 35 cycles of 1 min at 94°C for denaturation and extension at 72°C for 1 min. This was followed by a final extension at 72°C for 5 min. Primer sequences were shown in Table 1. Quantification was normalized using Glyceraldehyde 3-phosphate dehydrogenase (GAPDH) as an internal control. PCR products were then run on 2% agarose gels (Agarose I, AMRESCO, UT, USA) with SYBR safe (TTDNA01, BIOTOOLS Co., Taiwan) staining and images were analyzed using Mutigel-21 (Fluorescent Gel Image System TOP BIO Co., Taiwan). Images were analyzed using FloGel-I (Fluorescent Gel Image System, Top BioCom, Taiwan).

**Table 1:** Primer sets for PCR amplification.

Genes	Primer sequences (5’→3’)
HPV-16 E6/E7	ATG CAT AGT ATA TAG AGA TGG GAA T CTG CAG GAT CAG CCA TGG TAG A
Vimentin	CTA CAT CGA CAA GGT GCG CTT TGC CAG AGA CGC ATT GTC AA
α-SMA	CCA GCT ATG TGA AGA AGA AGA GG GTG ATC TCC TTC TGC ATT CGG T
human basic (h1) Calponin	GAG TGT GCA GAC GGA ACT TCA GCC GTC TGT GCC CAA CTT GGG GTC
Androgen receptor	CAT GCA CAA GTC CCG GAT G GGT GAG CTG GTA GAA GCG T
Interlukin1β	AAA GCT TGG TGA TGT CTG GT TCT ACA CTC TCC AGC TGT AG
IL-6	GGT ACA TCC TCG ACG GCA TCT GTG CCT CTT TGC TGC TTT CAC
IL-8	ATG ACT TCC AAG CTG GCC GTG GCT TCT CAG CCC TCT TCA AAA ACT TCT C
COX2	TTC AAA TGA GAT TGT GGG AAA ATT GCT AGA TCA TCT CTG CCT GAG TAT CTT
iNOS	GAT GAG AGT GGC AGC TAC TGG GTC TCC GCA CAA AGC AGG GCA CTG GGT C
TLR4	TGG ATA CGT TTC CTT ATA AG GAA ATG GAG GCA CCC CTT C
GAPDH	GCT CTC CAG AAC ATC ATC CCT GC CGT TGT CAT ACC AGG AAA TGA GC

### Western blotting

Cells were trypsinized and lysed on ice in RIPA buffer (50 mM Tris pH 7.4, 150 mM NaCl, 0.5% DOC, 1% NP-40, 0.1% SDS), followed by centrifugation for 15 min at 12,000 rpm at 4°C. The upper fluid, containing total protein, was collected and quantified. The equal amounts of protein was separated in 10% SDS-PAGE and blotted on a nitrocellulose membrane. The membrane was treated with blocking-buffer for 2 hours and incubated with primary antibodies at 4°C overnight. β-actin or lamin B was as an internal control. After brief washing in PBS-T, the membranes were incubated with peroxidase-labeled secondary antibody (diluted 1:5000 in PBS-T) for 2 h at room temperature. Protein bands were detected by the ECL plus-Kit (Amersham Pharmacia) and visualized with a Biospectrum AC Imaging System (UVP BioImaging Systems, Upland, CA). Primary antibodies included anti-AR (diluted 1:1,000, Genetex), anti-iNOS (diluted 1:500, Genetex), anti-p65 (diluted 1:1,000, Genetex), anti-human β-actin (diluted 1:5,000, Millipore), and anti-human lamin B (diluted 1:1,000, Cell signaling).

### Immunocytofluorescence (IFC) staining

IFC staining was performed to demonstrate that stromal cells were mixed population. Cells were fixed with 80% chilled methanol and washed twice in PBS, followed by a blocking step using Avidin/Biotin blocking kit (Vector Laboratories, Burlingame, CA) for 20 min. Immunoglobulin reactions were carried out using rabbit anti-human α-SMA monoclonal antibody (myofibroblast marker; diluted 1:200, Genetex), rabbit anti-human vimentin monoclonal antibody (fibroblastic marker; diluted 1:200, Genetex), and calponin (smooth muscle cell marker; diluted 1:100, Genetex) overnight as the primary antibody and anti-rabbit IgG secondary conjugated with Dylight 488 for 30 min at room temperature. After incubation with antibodies, cells were then reacted with DAPI and finally observed using fluorescence microscope. Images were acquired and processed using DPC controller software (Olympus, Hamberg, Germany).

### Tumorigenicity assay

The animal experiment was conducted in compliance with the protocol approved by the Institutional Animal Care and Use Committee of Taipei Medical University. The suspended mixture was injected subcutaneously into the dorsa of each SCID/NOD mice (6 mice per group), 4 to 6 weeks old, was provided by the National Taiwan University Laboratory Animal Center (Taipei, Taiwan). The mice were maintained in sterilized pathogen-free (SPF) cages and observed daily for tumor formation over 3 months. The primary hPSC and immortalized hPSC cells were trypsinized from confluent monolayer cultures and re-suspended at 4 × 10^6^ cells/mL in PBS for injection to mice. HeLa cells were injected as positive controls.

### Creation of chronic inflammation model in ihPSC and inhibited by hylan GF-20

ihPSC was finally subjected to study the inflammation mechanism related to chronic prostatic inflammation. Briefly, to create an *in vitro* model featuring chronic prostatic inflammation, ihPSC was stimulated with 20 ng/ ml IFN-γ and IL-17 for 48 hrs [17]. The high molecular weight hyaluronic acid (HA) hylan G-F 20 with strong anti-inflammatory potentials [20] was further treated inflammatory ihPSC for another two days. Cell proliferation, RT-PCR and western blotting were then utilized to evaluate the inflammation and anti-inflammation responses in ihPSC.

### Statistical analysis

The results of all experiments are shown as the mean ± standard deviation (SD). In the characteristic results, differences between parental cells and ihPSC were compared using student *t-test*. In the inflammation model, differences between control and treatment groups were compared and evaluated using student *t-test*. A *p* value < 0.05 was considered as a significant difference.

## Results

**Fig 1:**
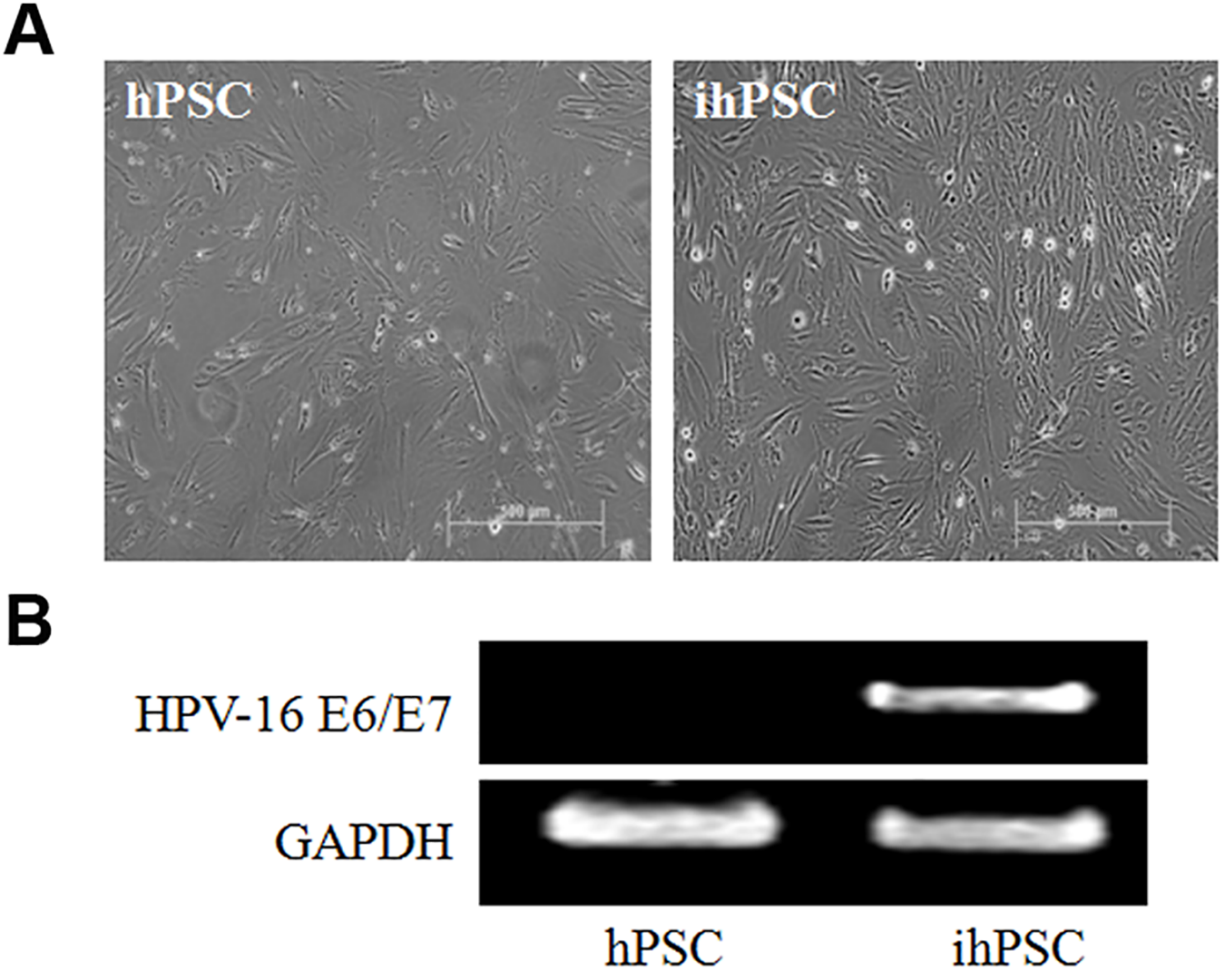
Characterization of HPV16 E6/E7-immortalized human prostate stromal cells (ihPSC). (A) No significant difference in morphological appearances of fibroblast-like cells between primary hPSC and ihPSC. Scale bar, 500μm. (B) Successful immortalization was confirmed by stable expression of HPV-16 E6/E7 mRNA in ihPSC cells by RT-PCR. Glyceraldehyde 3-phosphate dehydrogenase (GADPH) was used as internal control.

Human prostate stromal cells (hPSC) actively contributed to organ-specific inflammatory process in benign prostatic hyperplasia (BPH). However, primary prostate stromal cells rapidly lose its phenotypes during passages, especially the decreased expression of androgen receptors (AR), which exert an essential role in inducing the inflammatory responses [21, 22]. For preclinical studies, we attempted to immortalize primary hPSC by transduction of HPV-16 *E6/E7* retroviral vector (LXSN16E6E7). No significant difference was found in fibroblast-like morphology between primary hPSC and immortalized hPSC (ihPSC) by phase contrast microscopy (Fig 1A). Successful immortalization was confirmed by stable expression of HPV-16 *E6/E7* mRNA in ihPSC cells by RT-PCR (Fig 1B).

**Fig 2:**
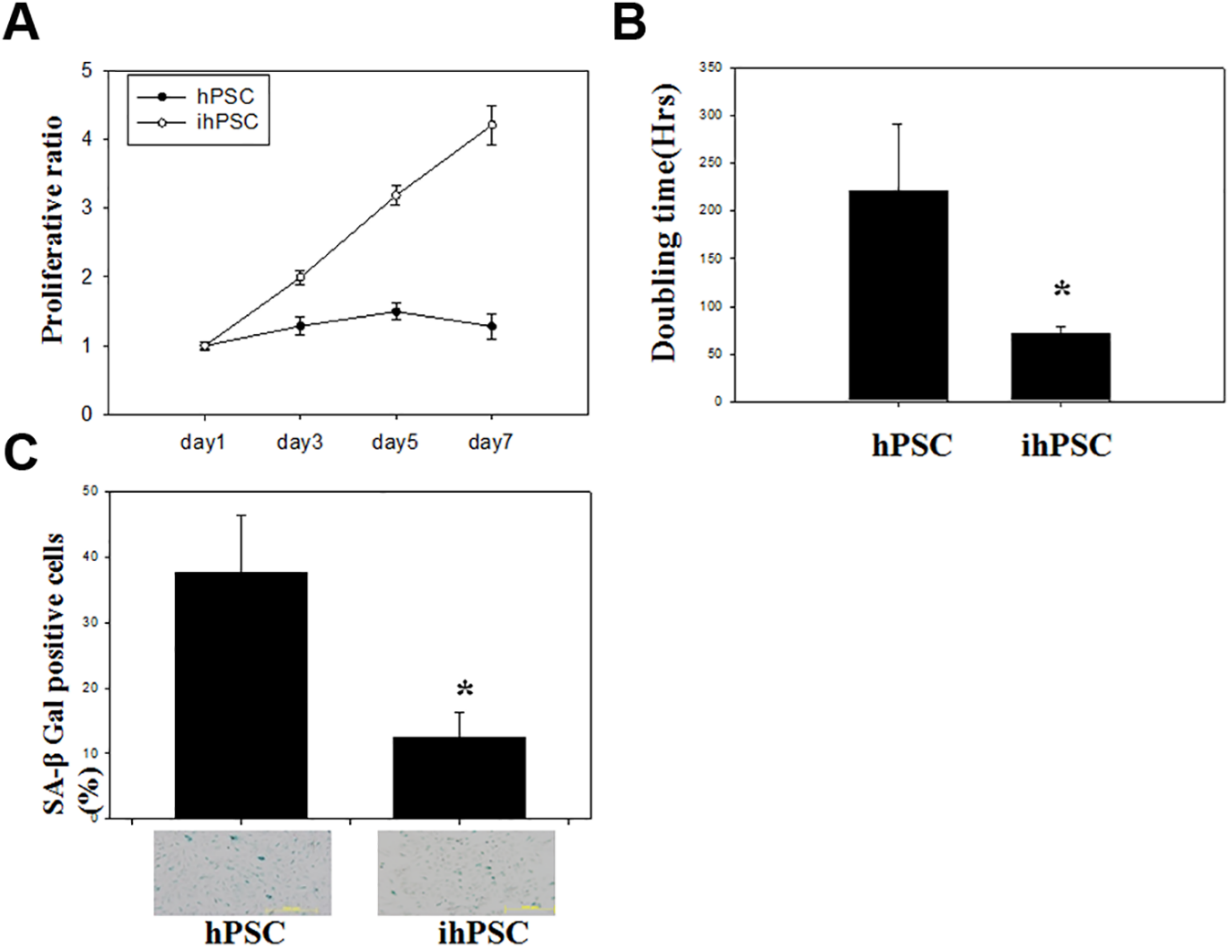
Cell growth and senescence of ihPSC. (A) A representative growth curve, (B) population doubling time (hrs), and (C) Senescence associated β-galactosidase (SA-β-gal) activity in ihPSC were compared to hPSC. Results are shown as the mean ±SD for three independent experimental cultures. * indicates a significant difference with *P*<0.01.

Proliferative characteristics of ihPSC were demonstrated by viability assay for 7 days (Fig 2A). The growth curve of ihPSC was distinct from primary hPSC, showing an arithmetic ratio of growth up to the 7^th^ day, while decreased proliferative ratio was observed in the primary hPSC from day 3. The ihPSC showed a significantly shorter cell doubling time (72.07±6.85 hrs) than the primary hPSC (220.4±71.43 hrs) (*P*<0.01, Fig 2B). Cell senescence of the ihPSC (12.48±3.75%) characterized by senescence-associated beta-galactosidase (SA-β-gal) activity was lower than that of primary hPSC (37.64±8.88%, P<0.01), indicating a significant decrease in the amount of senescent cells (Fig 2C). These results suggested that ihPSC possessed a significantly higher proliferation rate and anti-senescence compared to primary hPSC.

**Fig 3:**
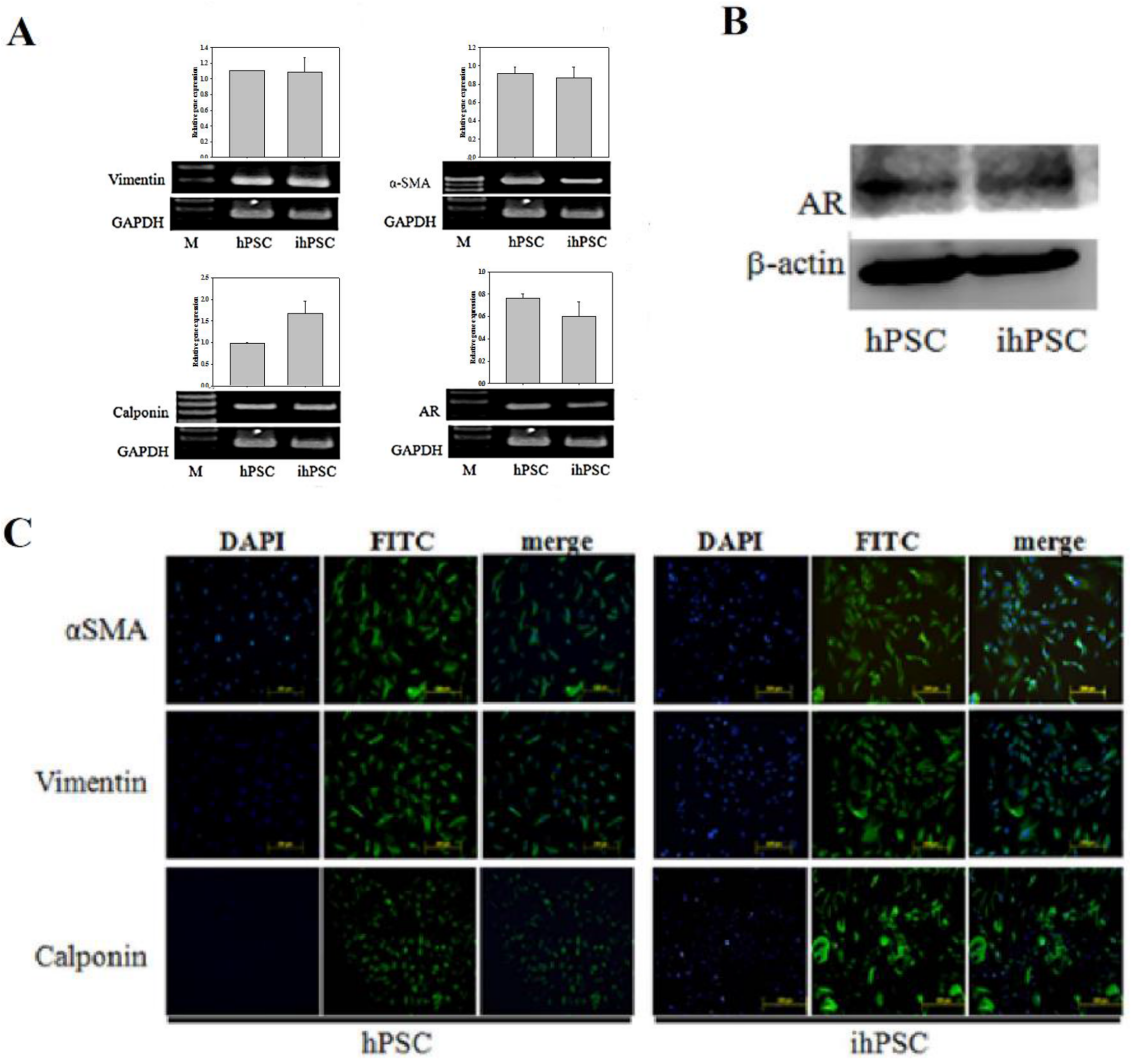
Phenotypic characteristics of ihPSC. (A) RT-PCR analysis of specific mRNA transcripts of cell markers, including vimentin, α-smooth muscle actin (α-SMA), calponin, and androgen receptor (AR) and their relative quantification. GADPH was used as internal control. (B) Western blot analysis for proteins isolated from hPSC and ihPSC cells confirmed the expression of AR with anti-AR antibody. β-actin was used as the internal control. (C) Immunocytofluorescence staining for α-SMA, vimentin, and calponin (using FITC labeled antibodies). Nuclei were counterstained with DAPI (blue). FITC and DAPI stainings were merged to show the localization of specific proteins. Scale bar, 200 μm. No significant difference was observed among cell markers during RT-PCR analysis.

After 25 passages of ihPSC culture, the markers of cytoskeleton (α-SMA and vimentin) and smooth muscle (calponin), especially the androgen receptor (AR) were examined and found continuously expressed in ihPSC, almost identical to the primary hPSC (Fig 3A). In Fig 3B, the protein level of AR by western blot between the primary hPSC and ihPSC expressed equally. Representative merged images of immunocytofluorescence (ICF) staining for α-SMA, vimentin, and calponin in ihPSC also revealed no observable significant difference compared to primary hPSC (Fig 3C). These results indicated that after immortalization the ihPSC still maintained the primary characteristics of fibroblasts (vimentin-positive), myofibroblasts (α-SMA-positive), and smooth muscle cells (calponin-positive).

**Fig 4:**
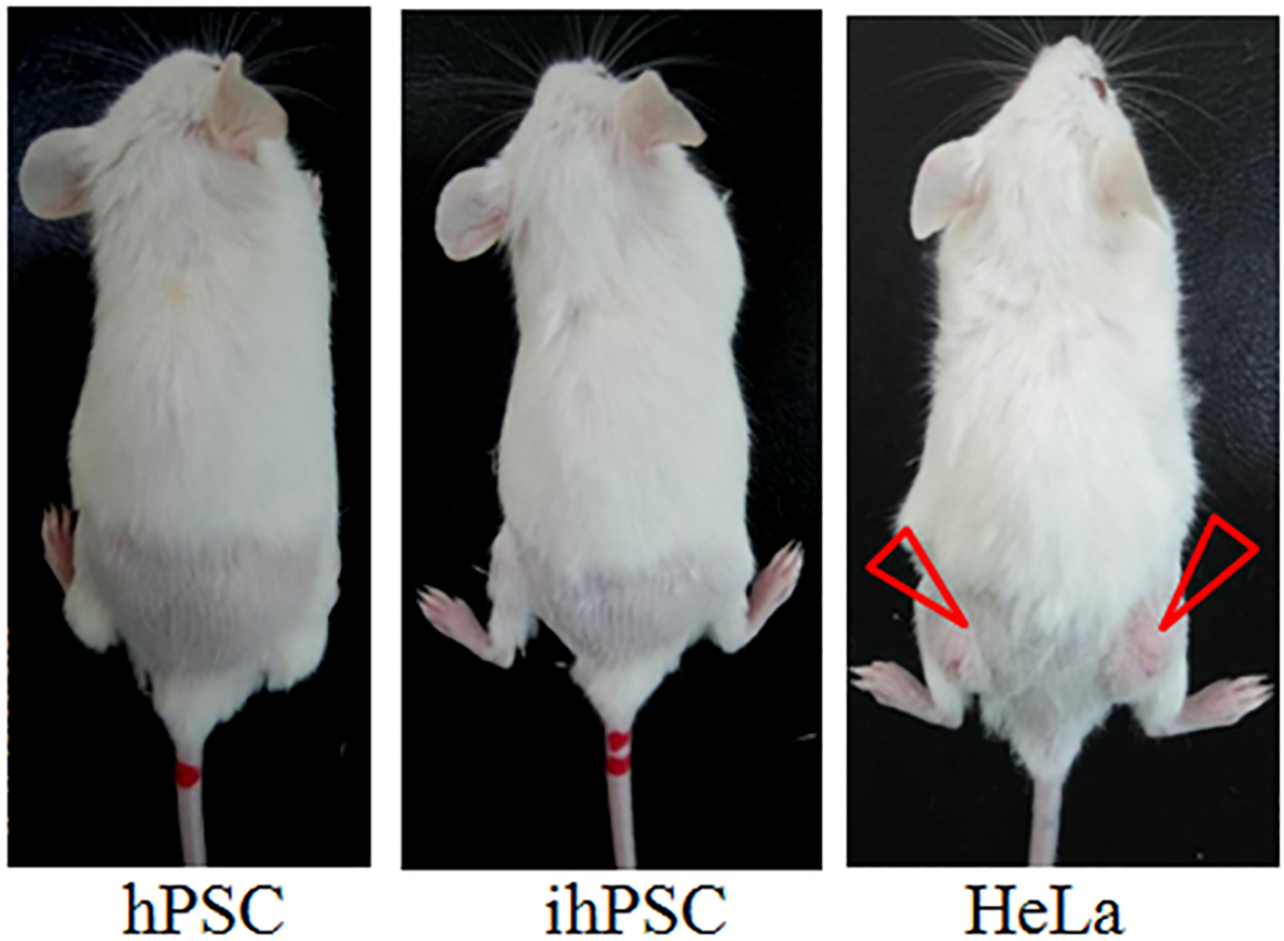
Tumorigenicity assay of ihPSC. NOD/SCID mice were subcutaneously injected with hPSC, ihPSC, and Hela cells (8 x 10^6^ cells/ mL). Tumor masses were only found on the dorsa of mice injected with HeLa cells (arrow indicated).

To examine whether HPV-16*E6/E7* genes induced tumorigenicity of ihPSC, the primary hPSC, ihPSC, and Hela cells (8 x 10^6^ cells/ mL) were injected subcutaneously into the dorsa of SCID/NOD mice (Fig 4). Three months later, tumor masses were only found on the dorsa of mice injected with HeLa cells (arrows indicated); however, both primary hPSC and ihPSC showed no tumor mass formation. These data indicated that both primary hPSC and ihPSC are both non-tumorigenic.

**Fig 5:**
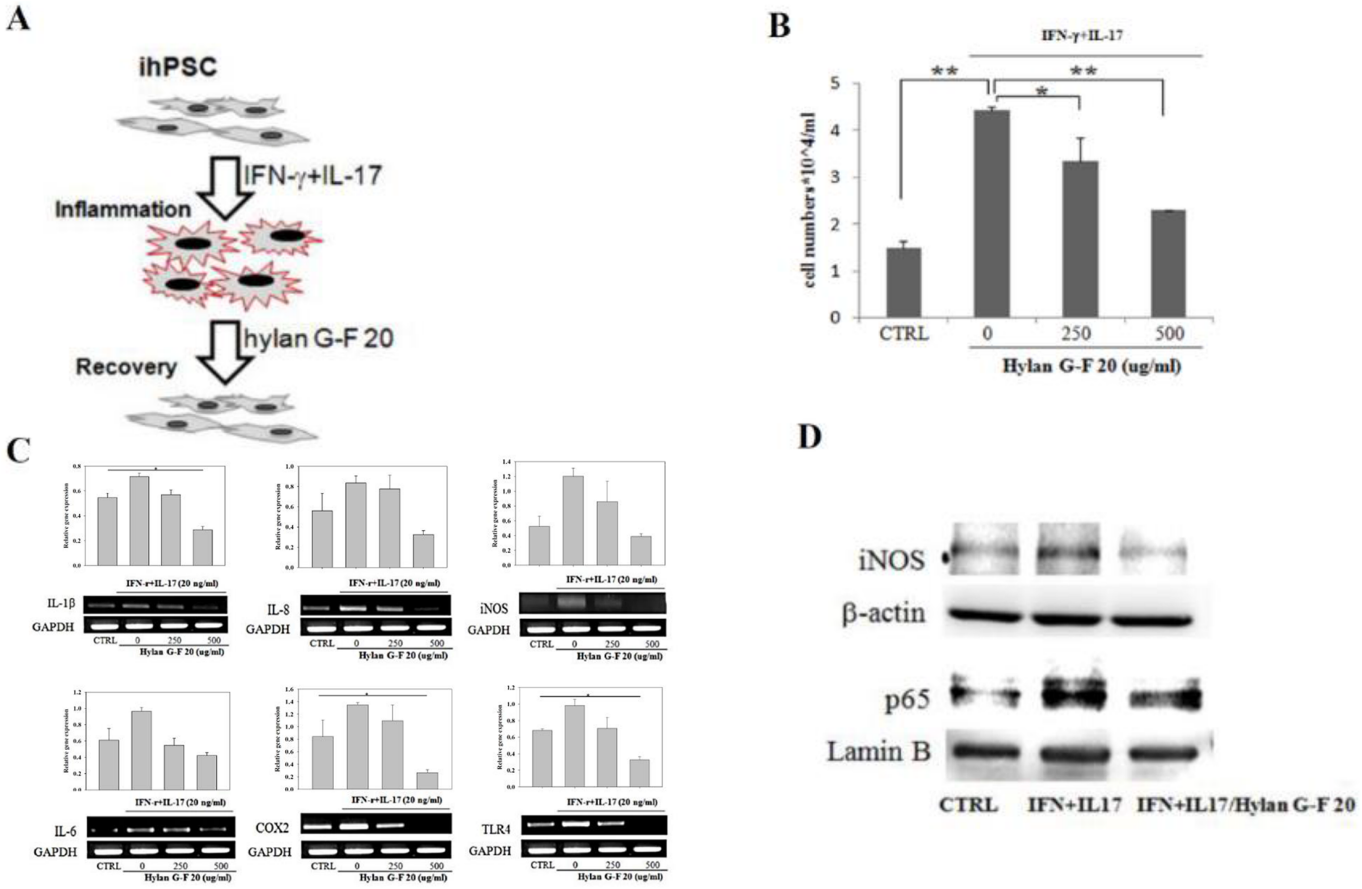
*In vitro* chronic prostate inflammation model in ihPSC induced by interferon-γ (IFN-γ) and IL-17. (A) Schematic illustration of *in vitro* chronic prostate inflammation model and anti-inflammatory effects of hylan G-F 20. (B) Cell proliferation, (C) RT-PCR analysis of prostate inflammation-associated markers and their relative quantification, GAPDH was used as internal control. (D) the expression levels of iNOS and p65 proteins (β-actin and lamin B used as respective internal control) in ihPSC cells treated with IFN-γ and IL-17 (20 ng/ml each) for 48h in the absence or presence of 250, and 500 μg/ml of hylan G-F 20 compared to the control (CTRL) group. Results are shown as the mean±SD for three independent experimental cultures. * and ** indicates a significant difference with *P*<0.01 and *P*<0.001, respectively.

We further investigated the important role of hPSC in chronic inflammatory process in BPH pathogenesis triggered by proinflammatory cytokines. For creating *in vitro* chronic BPH inflammation model, ihPSC were grown in the presence of IFN-γ+IL-17 (20 ng/ mL for each) [17] and then the effects of IFN-γ+IL-17 on the viability of ihPSC was determined after 48 h treatment (Fig 5A). The proliferation of ihPSC was significantly increased after IFN-γ+IL-17 treatment, suggesting that IFN-γ+IL-17-mediated inflammatory response had a significant impact on the growth of ihPSC (Fig 5B). However, the high molecular weight hyaluronic acid (HA) hylan G-F 20 could dose-dependently diminish the induced proliferation of inflammatory ihPSC.

To identify factors potentially responsible for the inflammatory effect of IFN-γ+IL-17 in ihPSC, the pro-inflammatory molecules were examined by RT-PCR (Fig 5C). Expression of IL-1β, IL-6, IL-8, cyclooxygenase 2 (COX2), inducible nitrogen oxide synthase (iNOS), and Toll-like receptor 4 (TLR4) were all increased by IFN-γ+IL-17 compared with untreated cells (CTRL). After treatment with G-F 20, the pro-inflammatory response was then almost totally diminished by higher concentration of hylan G-F 20 at 500 μg/ml. Interestingly, little effect was seen on IL-6 in hylan G-F 20.

During inflammation, iNOS and nuclear factor кB (NF-кB) subunit p65 cooperatively participated in the main inflammation process. This was evidenced by western blot analysis showing an increased expression of iNOS and p65 in response to IFN-γ+IL-17 in ihPSC compared to CTRL (Fig 5D). Such induction was effectively diminished by the treatment with hylan G-F 20 (500 μg/ ml). These results indicated that the inflammation phenotypes of ihPSC could be stimulated by IFN-γ+IL-17 and then down-regulated by hylan G-F 20.

## Discussion

In the present work, we attempted to establish an immortalized hPSC, designated as ihPSC, by transduction of HPV-16 *E6/E7*. After 25 passages, the ihPSC was first characterized according to the parental hPSC phenotypes. The ihPSC could stably expressed HPV-16 *E6/E7* gene and showed significantly increased proliferation rate, decreased senescence, and maintained parental phenotypes. In addition, ihPSC showed non-tumorigenic.

Primary human prostate stromal cells (hPSC) from BPH were shown to exhibit the key features of antigen presenting cells and produce pro-inflammatory cytokines and chemokines in response to the stimulation of IFN-γ and IL-17 [17]. However, primary cultures of human prostate stromal cells have limited lifespan and may lose their phenotypes during propagation. Thus, to overcome these limitations and to preserve a stable culture system for investigating clinically relevant problems, establishment of immortalized cell line mimicking the characteristics of primary cells has become an emergent strategy. Our previous results showed that the HPV-16 *E6/E7*-based approach has been utilized to successfully establish several immortalized platforms, including human articular chondrocytes (designated as hPi cells) [14] and human nucleus pulposus (designated as ihNP) [15]. Both cell lines can preserved stable chondrogenic phenotypes, in which the hPi cells can be used to induce the stage-specific chondrogenesis of MSCs and expressed the regenerative potential in platelet-rich plasma (PRP)/ collagen scaffold [13, 23]. In addition, the ihNP cells can provide a chondrogenic recovery model as a regenerative drug screening platform for further disc regenerative drug discovery. Thus, this study was to achieve the goal of obtaining stable hPSC cell line for prospective applications in prostate inflammation.

The parental hPSC was originated from a patient with BPH and concurrent prostatic inflammation. The stromal cell population in hPSC includes smooth muscle cells (calponin-positive), fibroblasts (vimentin-positive) and myofibroblasts (α-SMA-positive) [24]. It is known that the principal prostate stromal cells (smooth muscle cells and fibroblasts) undergo a phenotype switching to emerge as myofibroblasts [25]. Our results showed that in ihPSC, the mixed stromal markers including vimentin, α-SMA, and calponin were also confirmed indicating the stable phenotypes of ihPSC.

Another critical characteristic of hPSC is the expression of androgen receptor (AR), which assisted in promoting stromal cell growth and proliferation in prostate. Androgen also directly regulates prostate development and growth via androgen receptor [26, 27]. In addition, stromal AR could enhance the infiltration of macrophage migration toward prostate stromal cells in promoting BPH development [28]. Our results revealed that AR was strongly expressed in both ihPSC and parental hPSC (Fig 3). Hence, this indicates that ihPSC could be utilized as an *in vitro* stromal inflammation model of BPH. IFN-γ and IL-17 were up-regulated in BPH [6, 29], which leads to the hypothesis that BPH was proposed to represent an “immune inflammatory” disease. Consistent with a previous study using primary BPH cells, up-regulated IL-6 and IL-8 in ihPSC might be triggered with IFN-γ+IL-17 (Fig 5). The promoted proliferation of ihPSC induced by IFN-γ+IL-17 was associated with increased IL-8, which has also been proposed as the link between chronic prostate inflammation and autocrine/ paracrine stromal cell proliferation [8, 17]. Studies suggest that IL-6 is produced not only by prostate cancer epithelial cells but also by stromal cells, and is elevated in patients with metastatic prostate cancer and seems to mediate survival [30, 31]. COX-2 was found to be correlated in BPH and up-regulated within prostate cancer with chronic inflammation [32, 33]. iNOS has an increased expression in prostate cancer and BPH cells while TLR4 are associated with the alteration of innate immunity and inflammation in prostate cancer [34, 35]. NF-κB/ p65 expression was also significantly increased in experimental BPH, correlated to the pathologic proliferation of prostatic glandular and stromal tissues [36]. Taking together, currently the established ihPSC cell model could illustrate these inflammation-associated factors that may contribute to the activation of fibroblasts and infiltration of immune cells, which subsequently generate the microenvironment associated with the development of prostate inflammatory lesions.

In clinical practice, anti-inflammation strategies for BPH inflammation have been widely developed, including the uses of non-steroidal anti-inflammatory drugs (NSAID), anti-oxidant compounds and vitamin D receptor (VDR) agonists [37], however, efficacies were still limited. For alternative therapy, high molecular weight hyaluronic acid (HMW-HA) has been reported to reduce inflammation through blocking the induction of inflammatory signaling [38]. The sulfated HA also suppressed the growth and the anti-proliferative effects in prostate cancer cells due to the inhibition of hyaluronidase activity [39]. Hence, the HMW-HA hylan G-F 20 with strong anti-inflammatory potentials [20] was further utilized for treating the IFN-γ+IL-17-triggerred inflammatory ihPSC. Our results demonstrated that hylan G-F 20 could inhibit the inflammation responses in inflammatory ihPSC, including the reduced cell proliferation, inflammation phenotypes and inflammatory signaling. Since hylan G-F 20 has exerted its anti-inflammatory effects on osteoarthritis (OA) [40, 41], we are the first to examine its effects on BPH inflammation. It is reported that inflammation-induced PGE_2_ and NO concentrations could be suppressed by hylan G-F 20 [42]. Hylan G-F 20 can also interact with membrane receptor CD44 and intracellular adhesion molecule-1 (ICAM-1) for modulating the inflammatory effects, including inhibiting the IL-induced matrix-degrading enzymes and production of proinflammatory mediator, and also preventing cell apoptosis [43]. Collectively, hylan G-F 20 could potentially be further used for BPH treatment.

In summary, the ihPSC could provide a mechanism-based platform for investigating prostate inflammation. The ihPSC exhibited the major phenotypic features of primary hPSC and could undergo inflammatory response to pro-inflammatory cytokines (IFN-γ and IL-17) at transcriptional and translational levels. It could be a promising approach to study the potential therapeutic and protective effect of anti-inflammatory agents. Further, the HMW-HA hylan G-F 20 showed strong anti-inflammatory effects by decreasing inflammatory cytokines and signaling in the ihPSC, indicating its therapeutic potentials in BPH treatment in the future.
